# Improved Productivity with Multilaser Rotary Powder Bed Fusion Additive Manufacturing

**DOI:** 10.1089/3dp.2022.0288

**Published:** 2024-02-15

**Authors:** Peter Wang, Gordon Robertson, Brian T. Gibson, Chris M. Fancher, Jay Reynolds, Michael Borish, Jesus R. Cruz, Phillip Chesser, Benjamin Stump, Amiee Jackson, Eric MacDonald

**Affiliations:** ^1^ Manufacturing Science Division, Oak Ridge National Laboratory, Oak Ridge, Tennessee, USA.; ^2^ Material Science and Technology Division, Oak Ridge National Laboratory, Oak Ridge, Tennessee, USA.; ^3^ The University of Texas at El Paso, El Paso, Texas, USA.; ^4^ Computational Sciences and Engineering Division, Oak Ridge National Laboratory, Oak Ridge, Tennessee, USA.

**Keywords:** additive manufacturing, rotary powder bed fusion, mass production

## Abstract

Laser powder bed fusion (LPBF) enables the fabrication of intricate, geometrically complex structures with a sufficiently fine surface finish for many engineering applications with a diversity of available feedstock metals. However, the production rate of LPBF systems is not well suited for mass production in comparison to traditional manufacturing methods. LPBF systems measure their deposition rates in 100's of grams per hour, while other processes measure in kilograms per hour or even in the case of processes such as forming, stamping, and casting, 100's of kilograms per hour. To be widely adopted in industry for mass production, LPBF requires a new scalable architecture that enables many orders of magnitude improvement in deposition rate, while maintaining the geometry freedom of additive manufacturing. This article explores concepts that could achieve as much as four orders of magnitude increase in the production rate through the application of (1) rotary table kinematic arrangements; (2) a dramatic number of simultaneously operating lasers; (3) reductions of laser optic size; (4) improved scanning techniques; and (5) an optimization of toroidal build plate size. To theoretically demonstrate the possibilities of production improvements, a productivity analysis is proposed for synchronous reluctance motors with relevance to the electric vehicle industry, given the recent increase in the diversity of printable soft magnetic alloys. The analysis provides insights into the impact of the architecture and process parameters necessary to optimize rotary powder bed fusion for mass production.

## Introduction

Powder bed fusion (PBF) has become one of the most widely adopted additive manufacturing processes for the production of aerospace and biomedical products that require customized and/or complex structures fabricated from a wide variety of metal alloys.^1–3^ Numerous investigations into the productivity and cost of traditional laser PBF (LPBF) have been explored,^3–7^ as both are impediments to broader adoption by industry for the traditional Cartesian LPBF architectures. These systems are based on linear motion orchestrated in three orthogonal axes: a structure is built layer by layer in the Z-axis; a recoater works in the X-axis; and gas flows generally in the Y-axis, as defined by the ISO/ASTM 52900 standard,^8^ all of which are confined to a rectangular build volume.

Two mutually exclusive operations are required for each layer of powder—a recoater spreading the powder and a laser melting the powder. These two operations result in periods of inactivity for the laser, and consequently, reduced laser duty cycle—impacting the productivity of the process. The production rate of Cartesian systems can be improved by scaling to larger build volumes and by increasing the number of lasers^3,9^; however, the scan path stitching in multilaser systems increases the complexity and control of the thermal history and requires precise scan calibration.

Early investigations of rotary architectures were proposed by Hauser C et al.^10^ and termed “Spiral Growth Manufacturing,” recognizing the possibility of continuous laser melting with a polar coordinate system, with a toriodal powder bed. This early work was confined to small rotating powder beds that simultaneously performed powder recoating and laser melting—thus improving laser utilization. In addition, investigations in parallel productivity have found that gains exist in other forms of additive manufacturing, including directed energy deposition, in which continuous spiral or helical toolpaths have been demonstrated^11–15^ to improve productivity.

However, these are less impactful due to a single feedstock deposition and melting step rather than two mutually exclusive processes in LPBF. The rotary LPBF concept was later pursued with a more ambitiously large toroidal build platform targeting the optimized fabrication of large cylindrical structures often necessary in the aerospace industry, such as jet engines.^16^ Possible advantages that have been identified for such rotary LPBF include the simultaneous fabrication of multiple build layers, smaller fields of view of laser optics as the powder bed is in motion, and improved management of inert gas flow.^5^

If the powder bed cannot be lowered for each layer, a containment wall must be built enclosing the intended structure—as opposed to a permanent bed container in the Cartesian architecture, which has a descending floor. The containment wall in a rotary system can conform to the fabrication geometry, as powder dispensing is independent of the difference between the outer and inner diameter, and consequently, significantly less unmelted powder is required. However, this rotary architecture introduces increased complexity by requiring (1) “mark-on-the-fly” selective laser melting for the moving powder bed, with the “mark” terminology referring to laser marking on a solid substrate (e.g., serial numbers, etc.) with the substrate in this additive manufacturing case being a powder bed; (2) and the geometric considerations of the polar coordinate system (e.g., motion is faster on the outer edge when compared to the inner edge of the toroidal powder bed).

**FIG. 1.**
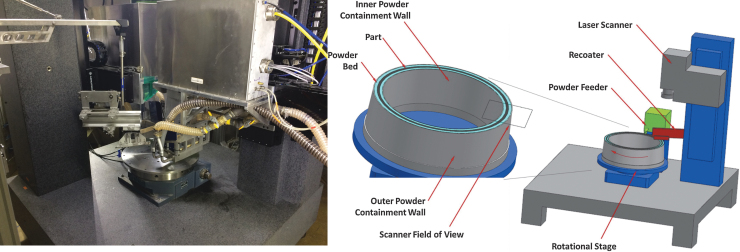
Rotary PBF system located at Oak Ridge National Laboratory (*left*) and the schematic of the original system at GE Global Research^16^ (*right*). PBF, powder bed fusion.

This article investigates the impact on production rate of components of rotary PBF to understand how these systems can enable the most industrially adopted additive manufacturing process for purposes of mass production. As slow production rates have largely impeded the broad adoption of PBF, the hypothetical productivity enhancements of rotary PBF are explored in this article. The study focuses on a mock reluctance motor design that is relevant to the electric vehicle industry—particularly in light of advancements in magnetic material development for general PBF. By exploring the toroidal build volume and the theoretical number of powder dispensers and lasers, a comparison of Cartesian and Polar architectures is discussed and the fundamental limits in productivity are elucidated. Figure 1 illustrates the Rotary LPBF system originally developed at General Electric Global Research and subsequently transferred to Oak Ridge National Laboratory and serving as the target system for this productivity analysis.

## Materials and Methods

The following section describes an existing system, and the methodology for estimating and comparing production rates for both traditional Cartesian and Rotary LPBF for a mock synchronous reluctance motor. The analysis accounts for build chamber shape (rectangular vs. toroidal), build chamber size (width/length or inner/outer diameter), and the number and power of lasers.

### The Rotary PBF

This rotary PBF system has two independent lasers and optics trains in an ascending head configuration over a 1 m diameter rotary build table. In this configuration, the laser optics move upwards as the layers are deposited, while the toroidal table rotates the build plate and does not move in the vertical direction. As the part rotates under the field of view, the laser will melt the powder. This configuration is better for building tall and heavy components. Tall components will significantly magnify any base plate misalignment, so a vertically stationary rotary table will reduce this error.

However, translating the laser optics trains, powder feeding system, and recoater system vertically adds significant system complexity. In the descending bed configuration, the laser optics are stationary, while the rotary table moves downwards as layers are deposited. This configuration will work well for shorter builds and for very large diameters. This configuration significantly reduces the number of moving components, but adds the challenges of maintaining a level of a heavy build table. Neither case impacts this study.

### Reluctance motor as an exemplar application for productivity analysis

**FIG. 2.**
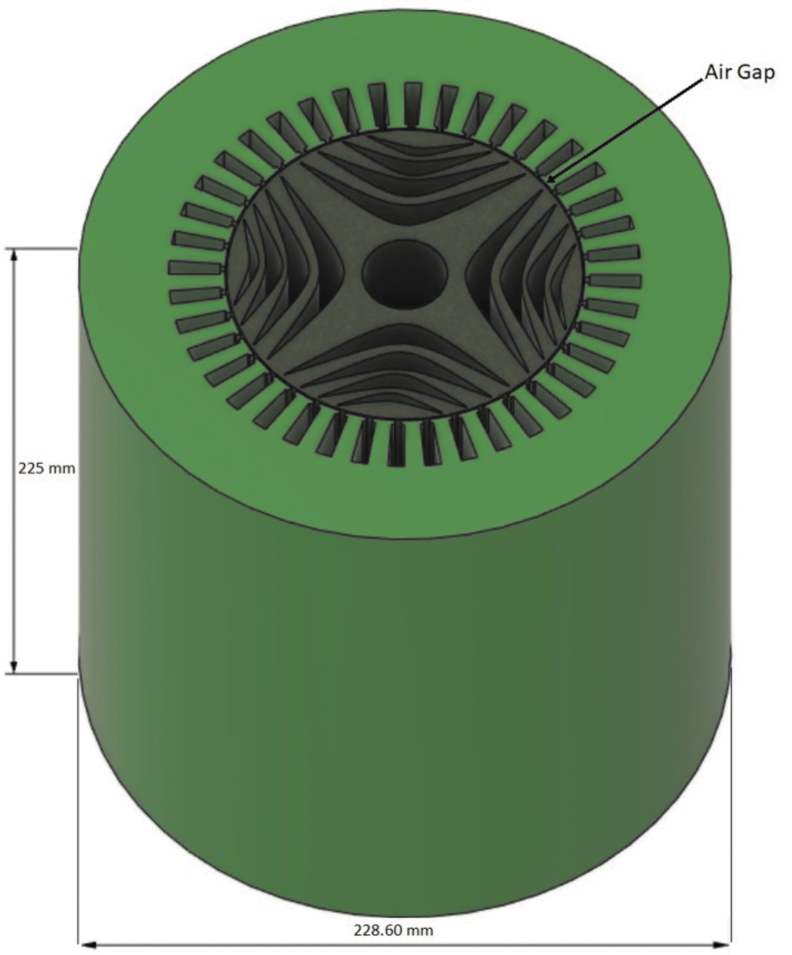
Notional rotor/stator design used for analysis. Dimensions in mm.

Assumptions of production requirements for this hypothetical case study include 300K vehicles produced in a quarter with two motors required per vehicle to approximate the production rate expected to be reached in the years following the publication of this article.^17^ The motor design in this work (Fig. 2) was notionally inspired by Ibrahim M et al.^18^ with a size relevant to electric vehicles as the exemplar application. The motor design includes the stator and rotor built together for an assembly 225 mm in both diameter and in height. Although the design has a constant cross-section, the two-piece fabrication includes substantial design complexity as well as the advantage of using topology optimization and machine learning to adapt the geometry digitally.

### Future cost and physical volume assumptions for lasers

**FIG. 3.**
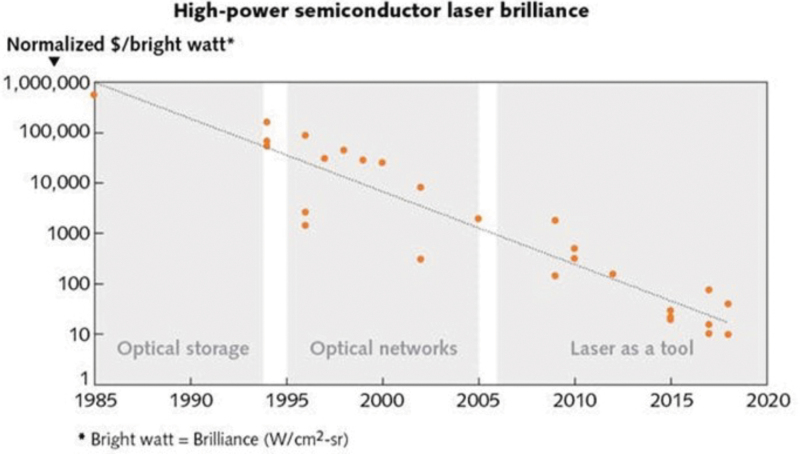
Laser cost versus time: a historical perspective.^19^

Currently, high-power lasers required for melting metal powder are expensive and large. However, similar to Moore's law in the semiconductor industry, lasers continue to improve exponentially in terms of performance and economics and this work assumes that the trend will continue with the ultimate consequence being that the laser time, placement, and cost for additive manufacturing (AM) systems will not be limiting factors restricting the number of lasers used for either Cartesian or Rotary architectures. Figure 3 depicts historical cost data for lasers with a compounding decrease per year. Although these data cannot be extrapolated into the future, high-power lasers are clearly becoming less expensive and smaller. While the feasibility of developing additive manufacturing systems with hundreds or even thousands of lasers may not be imminent, the possibility is likely inevitable.

### Productivity assumptions with Cartesian LPBF

The production rate for Cartesian LPBF is ultimately limited by the recoater time as this step per layer cannot be avoided, as opposed to the use of continuous Rotary architectures. The laser melting time for current systems dominates the build time, but this fraction of time could be reduced by using multiple and/or faster lasers^20^ and the lower bound rate for full production is dictated by the interlayer time (ILT), even if laser melting could be eliminated by completing each layer instantaneously.

Conventional Cartesian systems have an ILT in which powder is spread and the bed is prepared for the next layer. ILT was a term from Mohr et al., an article in which the term was defined as the time between each subsequent energy input into the build volume.^23^ ILT is useful in analyzing LPBF systems because many traditional systems have a non-negligible and difficult-to-eliminate ILT. Furthermore, the size of the powder bed determines how many motors could be packed simultaneously and ultimately establish the production rate in conjunction with the time per layer. Production time for Cartesian LPBF is consequently captured by Eq. (1),

(1) $$\begin{array}{cc} & TotalParts\\ & =\left(\frac{\rho_{packing}*A_{table}}{A_{part}}\right)\frac{Time}{t_{plateChange}+\left[\frac{PartHeight}{LayerHeight}\right]*ILT} \end{array}$$

in which the leftmost fraction is the number of parts per build, with ρ_*packing*_ being the packing density of the motors, and *A_table_* and *A_part_* are the areas of the table and parts, respectively. The rightmost fraction is the number of builds completed in a specified amount of *Time*. *t_plate Change_* is the time needed to change the build plate on the table. *Part height* and *layer height* are the heights of parts and layers of powder, respectively. *ILT* is the ILT that is dedicated to recoating the next powder layer.

### Productivity assumption for an existing Rotary LPBF system

To establish the baseline state-of-the-art production rate for a Rotary LPBF system, a print was planned that includes the printing of nine sample parts (225 mm diameter, 225 mm tall reluctance motors), which is the maximum number of structures that can fit on the 1.0 m diameter build plate in a single toroid configuration (a packing efficiency of 0.46). This configuration allows for the part cross-sections to fit entirely within the fields-of-view of the two laser heads on the system, assuming the nominal scan field area (250 × 250 mm) is utilized. ORNL Slicer 2 software,^21,22^ which has capabilities for slicing fabrications specifically for multilaser rotary LPBF systems, was used to generate the build files for this proposed print, and then the files were post-processed to generate the timing and auxiliary build files that are necessary for operation.

**FIG. 4.**
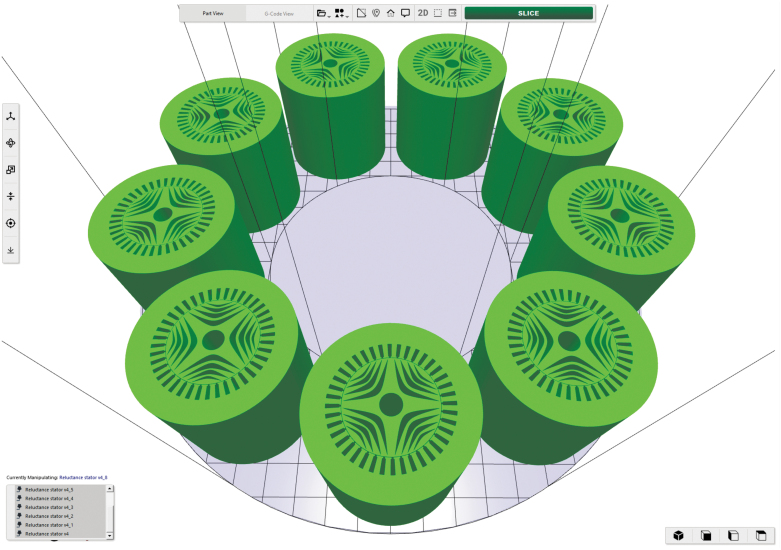
Nine sample parts loaded on a 1 m diameter build plate in ORNL Slicer 2.

This process uses accurate laser scan time estimation, which requires an accounting of the Scanlab RTC5 controller marking parameters, including laser on/off delays and selective melting delays, to determine the desired revolutions per minute (RPM) of the system, which is variable depending on the cross-section load being scanned, and to calculate the total print time for a build. Figure 4 illustrates the placement of nine reluctance motors analyzed by the slicer software for a bed with an outer diameter of 1.0 m and an interior toroid diameter of 0.5 m.

**FIG. 5.**
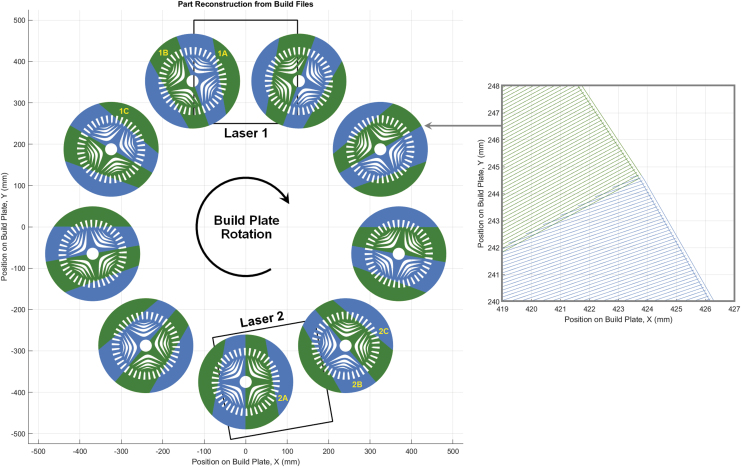
Sample part reconstruction; Laser 1 (*green*), Laser 2 (*blue*) with letters (A–C) depicting sectors that are selectively melted simultaneously (e.g., 1A and 2A are created coincidently).

The rotary LPBF system requires segmentation, or sectoring, of large part cross-sections to ensure that regions being selectively melted by the laser fully reside within the fields-of-view of the laser. ORNL Slicer 2.0 uses a methodology that subdivides the contents of the build plate into sectors (completely in the field of view of the optics), and for the print in question, 36 sectors were used. A reconstruction of the reluctance motor geometries from the build files is shown in Figure 5, with a zoom-in from the macro view to the scan path details for the first layer. An alternating scan strategy was employed, in which each laser is responsible for every other sector.

One caveat associated with the planning of this particular print was that an assumption was made that laser 2 could be positioned at an ideal location (170° from laser 1, rather than diametrically opposed), such that good load balancing in laser melting could be achieved without further manipulation of build files. Figure 5 shows examples of sectors that are melted simultaneously in pairs for this two laser system example (e.g., sectors 1a and 2a, sectors 1b and 2b, etc.).

The parts were sliced, and print times were computed using the parameters listed in Table 1. A range of scan speeds (1000–4000 mm/s) was used to determine the impact of speed on laser productivity; scan speed, along with layer height and hatch spacing, is the parameter of significant influence on total system productivity. A scan speed of 1000 mm/s has been used for multiple test prints with Inconel 718 powder on this system, but beyond that, no claim is made as to the efficacy of parameter combinations with higher scan speeds, and values for laser power and spot size are not included in Table 1 as these parameters do not directly influence the calculation of print time.

**Table 1.** Existing Two-Laser Rotary Laser Powder Bed Fusions Slice and Print Time Computation Parameters

Parameter	Value
Layer height (μm)	50
Number of layers	4500
Number of sectors/layer	36
Scan speed (mm/s)	1000–4000
Hatch and perimeter spacing (μm)	120
Number of perimeters	2
Infill overlap w/perimeter (μm)	50
Infill hatch rotation/layer (°)	67
RTC5 marking parameters	
Laser on delay (μs)	200
Laser off delay (μs)	400
Mark delay (μs)	250
Jump delay (μs)	1000
Variable jump delay	Off
Polygon delay (μs)	200
Variable polygon delay	On
Edge level (μs)	400
Jump speed (mm/s)	30,000
Acceleration, estimated (mm/s^2^)	22,000,000

One final caveat is that brief periods of laser downtime between sectors, typically on the order of 200 ms for this system, were not considered in the calculations to minimize machine-specific properties and to obtain an estimation of the throughput that is inherent to each laser with the properties that are intrinsic to the sectors, that is, scan path and primary printing parameters.

### Productivity assumption for hypothetical LPBF: unlimited lasers

To determine the scalability of the number of lasers in a single Cartesian or Rotary system, a few key equations have to be delineated. The first of these is a measure of total parts produced over an arbitrary amount of time found by multiplying the number of cycles by the number of parts per cycle, as shown in Eq. (2).

(2) $$TotalParts=\frac{Time}{CycleTime}*\frac{Parts}{Cycle}$$

The *Cycle Time* in this equation can be broken down further by looking at the time needed to change a build plate, the total volume of powder being melted, the number of lasers melting the powder, and the throughput of those lasers. This is Eq. (3),

(3) $$CycleTime=t_{plateChange}+\frac{V_{parts}*\frac{Parts}{Cycle}}{\frac{A_{table}}{A_{optics}}*LaserProductivity}$$

in which *t_plate Change_* is the time needed to change a plate in the system, *V_parts_* is the volume of each part, *N_parts_* is the number of parts, and *A_table_* and *A_optics_* are the surface areas of the table and laser optics, respectively.

The optical area is the surface area above the print bed needed to mount an optical scanner and determines the upper limit for the number of lasers that can effectively work on the build table for either Rotary or Cartesian LPBF systems. A smaller optical scanner area means more lasers can be placed above the powder bed and one lower bounding assumption on build time of this present effort is the case in which the optical area approaches zero area, and consequently, the number of lasers that can be used in a single system simultaneously approaches infinity.

The *Laser Productivity* is the amount of powder each laser can melt—a combination of various factors such as laser power, scan speed, and layer height. The effective productivity of a laser will depend on the laser, the part geometry, and the hardware and software specifications of the laser, such as laser on/off delay, the slicing software used, and the laser scan speed. The *Parts/Cycle* is determined by the size of the parts, the size of the table, and how densely they can be packed onto the table. The following equation describes how many parts can be placed on the table.

(4) $$\frac{Parts}{Cycle}=\frac{\rho_{packing}*A_{table}}{A_{part}}$$

$\rho_{packing}$ is the packing density of the parts and *A_part_* is the area on the build plate each part takes up. This framework allows for a simulated estimate of a system output for an arbitrary part based on three key factors—laser productivity, table area, and optical laser scanner area.

Expanding Eq. (3), an element to account for the effect of the ILT was added for the specific case of Cartesian LPBF systems in Eq. (5). The value of ILT for Rotary systems was set to zero and eliminates the final term of the equation. A fabrication simulation of the reluctance motor was executed using these equations to analyze the quarterly output of a rotary powder bed system. The parameters of the simulation are shown in Table 2.

**Table 2.** Simulation Parameters

Parameter	Cartesian system value	Rotary system value
Part volume	0.007279 m^3^	0.007279 m^3^
Part diameter	225 mm	225 mm
Part height	225 mm	225 mm
Minimum distance between parts	1 mm	1 mm
Packing density	0.75	0.75
Plate change time	2 h	2 h
Hours per quarter	2190 h	2190 h
Range of laser throughput	0–600 cc/h	0–600 cc/h
Optical scanner area	25–475 cm^2^	25–475 cm^2^
ILT	5 s	0 s
Bed shape	Square	Toroidal

ILT, inter-layer time.

(5) $$\begin{array}{cc} CycleTime & =t_{plateChange}+\frac{V_{parts}*\frac{Parts}{Cycle}}{\frac{A_{table}}{A_{optics}}*LaserProductivity}\\ & +\left(\frac{PartHeight}{LayerHeight}\right)*ILT \end{array}$$

## Results and Discussion

To compare the production rate of AM systems, simulations of both Cartesian and Rotary systems were performed with the intention of observing how various factors impact the total quarterly production output. This analysis estimates how future improvements in PBF could impact LPBF mass manufacturing. Using these parameters, simulations were run in both ORNL Slicer 2 and Python to measure the parts per quarter of a theoretical system operating full time. ORNL Slicer 2 was used for simulating this two-laser rotary system. The python simulation uses the parameters in Table 2 as well as Eqs. (1–4) to simulate alternative system configurations that ORNL Slicer 2.0 is not configured to handle effectively, configurations with a large number lasers (i.e., hundreds or even thousands).

### Diminishing returns with increases in scan speed for both Cartesian and Rotary PBF

**FIG. 6.**
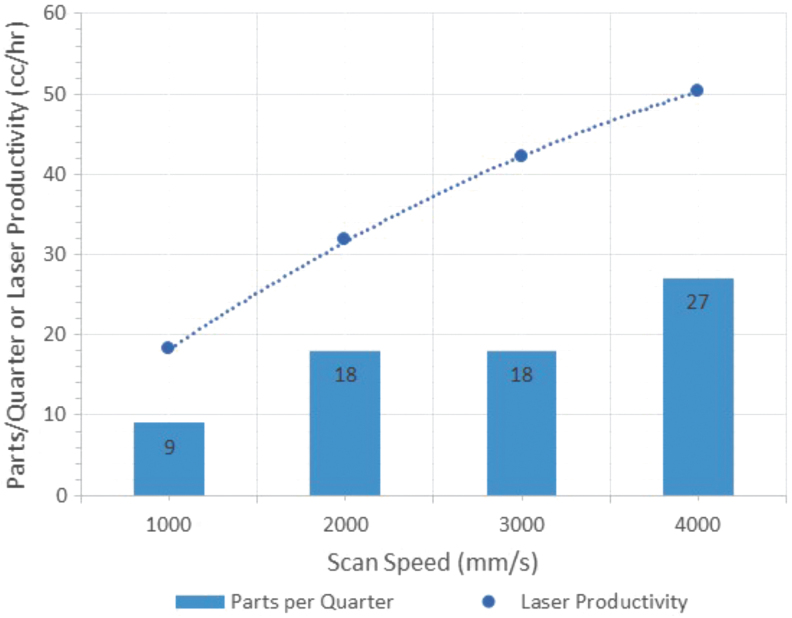
Nonlinear relationship between scan speed and laser productivity for the reluctance motor print on the existing Rotary PBF system, but with the same trend found in Cartesian systems; a 2nd-Order Polynomial Fit is shown.

The relationship between scan speed and laser productivity is not linear for LPBF systems even with the higher associated duty cycle (i.e., on-vs.-off time), as illustrated in Figure 6, regardless of the architecture (Rotary or Cartesian). The use of higher scan speeds yields diminishing returns for productivity because of the acceleration of the laser spot that is required within each vector, meaning that depending on vector length, the desired scan speed may not actually be achieved, a phenomenon that becomes more influential with higher scan speeds.

Clearly, laser scan speed increases only provide a less-than-linear impact on productivity and would require significant development and optimization to maintain yield and quality. Consequently, increasing scan speed is not the silver bullet required to obtain economically justified mass production with LPBF, for either Cartesian or Rotary, and is considered to be constant in this analysis over time to avoid reduction in part quality.

### Cartesian PBF productivity simulations

In the case of Cartesian architectures, the diminishing reward of laser scan speed is likely exacerbated as the ILT (not required in Rotary systems) forces a fixed minimum time for each layer, and any increase in laser speed cannot improve productivity further. To investigate the limiting effects of ILT in Cartesian systems, a simulation was run in which the laser build time was assumed to be instantaneous. That is to say, the only factors in determining the print time are the ILT and plate change time.

**FIG. 7.**
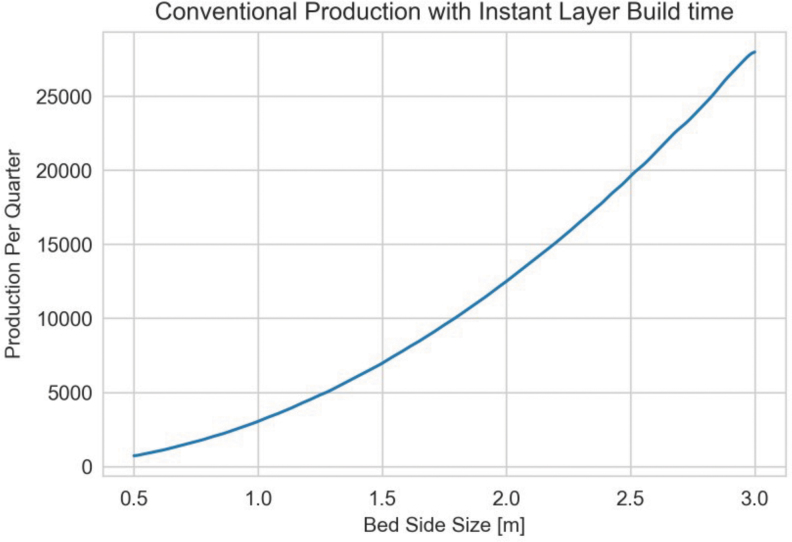
Quarterly production of a range of conventional Cartesian systems in which only ILT was considered (instantaneous selective laser melting with an infinite number of lasers). ILT, interlayer time.

**FIG. 8.**
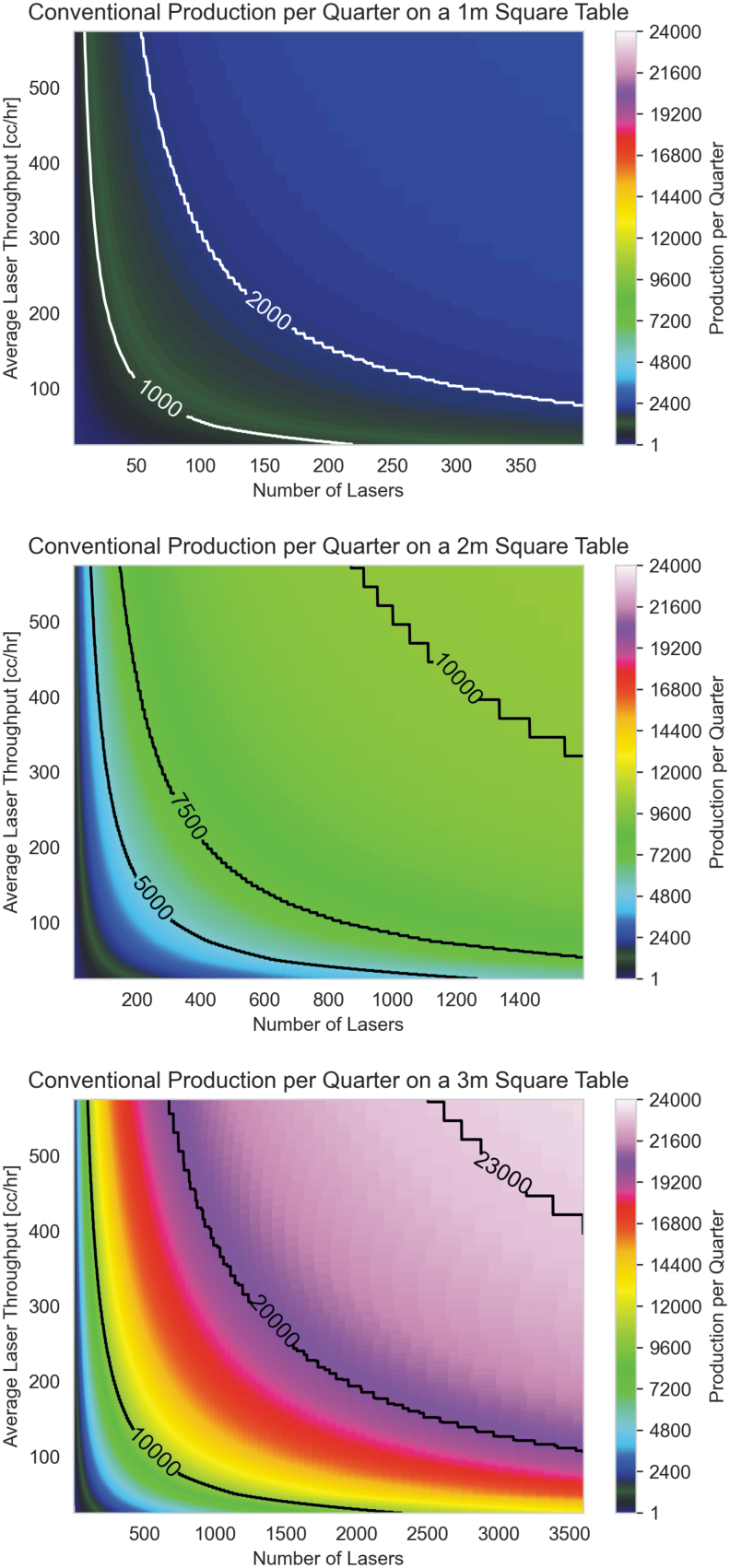
Quarterly production for Cartesian systems with a 1, 2, and 3 m squared bed.

Figure 7 shows the quarterly production of a range of Cartesian PBF systems with bed sizes ranging from one to three meters on a side to evaluate the impact of bed size for Cartesian systems. Even with an instant melt time per layer, the production rate is just slightly more than predicted by the simulation in Figure 8, which does incorporate the time required to melt each layer. Consequently, improving melting time provides a benefit that is slightly better than linear; however, the electric vehicle motor production example requires exponential improvements to both melting time and ILT to fully leverage additive manufacturing and eventually lead to broad industrial adoption.

The quarterly production of conventional laser powder bed system is limited by the requirement to recoat between each laser melting. This ILT immediately dominates the production time. A part that is only 225 mm tall with 50 μm layer height will have 4500 layers. With only a 5 s ILT, the system will spend over 56 h recoating. While ILT varies from machine to machine, analysis previously reported has consistently assigned an ILT value of upward of 10 s.^23–26^

For a conservative analysis when comparing Cartesian and Rotary systems, a 5 s ILT was used as a constant. Figure 8 shows simulations executed for quarterly production of a conventional system using Eq. (4) as the basis for cycle times. Note that due to the relatively low production rate, the laser throughput and quantity breakpoints for builds finishing are evident and cause a stair-stepping graph shape. A baseline case for conventional LPBF with a single system with a 1 m bed, two 50 cc/h lasers, and a 10 s ILT can produce a paltry 17 U per quarter.

From Figure 8, the lack of productivity is obvious in the context of a hypothetical production requirement of 600K motors quarterly. With current conventional large Cartesian LPBF systems, any one system could produce ∼17 U and thus would require operating ∼35,000 additive manufacturing systems to meet the quarterly rate. As these systems generally cost approximately a million USD and would require a full-time operator to operate, the possibility of employing current traditional systems exists but would likely be prohibitively expensive. If Cartesian systems could be improved through increases in the powder bed size and the number of lasers operating simultaneously, as hypothesized by Figure 8 bottom, an order of magnitude of improvement can be realized. However, even with 24K units produced per quarter, 30 of these large hypothetical systems would be required to meet the production rate.

### Productivity of the existing Rotary LPBF

The calculated results for total print time, average build plate RPM, total system productivity, and laser productivity, which is simply the total system productivity divided by the number of lasers, are shown in Table 3. Using a scan speed of 1000 mm/s yields a laser productivity of 18.17 cc/h, and the impact of increasing scan speed is apparent, with a laser productivity of 50.3 cc/h achieved with a scan speed of 4000 mm/s.

**Table 3.** Laser Productivity for Printing Nine Motors Using the Existing System

Scan speed (mm/s)	Print time (hours)	Average RPM	Productivity (cc/h)	Laser productivity (cc/h)	Parts per quarter
1000	1804.9	0.042	36.33	18.17	9
2000	1033.2	0.073	63.47	31.73	18
3000	788.0	0.096	84.29	42.14	18
4000	651.8	0.115	100.60	50.30	27

Note: volume of nine motors = 65,574 cc.

RPM, revolutions per minute.

The rotary powder bed system is not fully optimized with only two lasers currently. If the existing system were retrofitted with 20 lasers, the production rate could be increased quarterly as described in Table 4. This system description is based on the existing capabilities and limitations with this system. One of these major limitations is a low part packing density due to limited laser coverage. The system can only effectively print nine motors per build. However, with an optimization through an increase to 20 lasers (currently possible with the size of implemented lasers and the remaining space available above the bed), the build area would be increased by reducing the inner diameter and resulting in a much thicker toroidal build area.

**Table 4.** Current Part Production for the Optimization of the Existing Rotary System

	Existing two laser Rotary PBF	If improved to 20 lasers
Powder bed diameter	1 m	1 m
Laser productivity	50.3 cc/h	50.3 cc/h
Optics area	406 cm^2^	406 cm^2^
Number of lasers	2	20
Part diameter	225 mm	225 mm
Part height	225 mm	225 mm
Part packing density	0.46	0.75
Parts per quarter	27	294

PBF, powder bed fusions.

The larger build area enables much higher part packing densities. The addition of more laser optical trains above the build plate allows for parts to be fabricated at the center in tiers of the build area since the scanners can fully encompass that area. Such dense packing is not currently possible with only two lasers. Based on Eq. (4), this configuration relegates the system to printing a maximum of 14 motors per build. This configuration and optics train include sufficient space, to include up to 20 lasers and powder dispensers. With 20 lasers in the potential next generation rotary LPBF system, the number of motors jumps to 294 per quarter; however, even with this improvement, over a 1000 of these 20-laser systems would be required to meet production demand.

### Productivity of hypothetical Rotary with increased laser count and build volume

A rotary system has the potential to produce large quantities of parts per quarter as all lasers selectively melt powder on multiple build layers simultaneously with nearly full duty cycle. Unlike conventional systems, which are limited by their ILT, rotary bed systems do not have such a limitation, and increasing the number of lasers provides a translation of improved productivity improvements—almost linearly increasing the quarterly production.

**FIG. 9.**
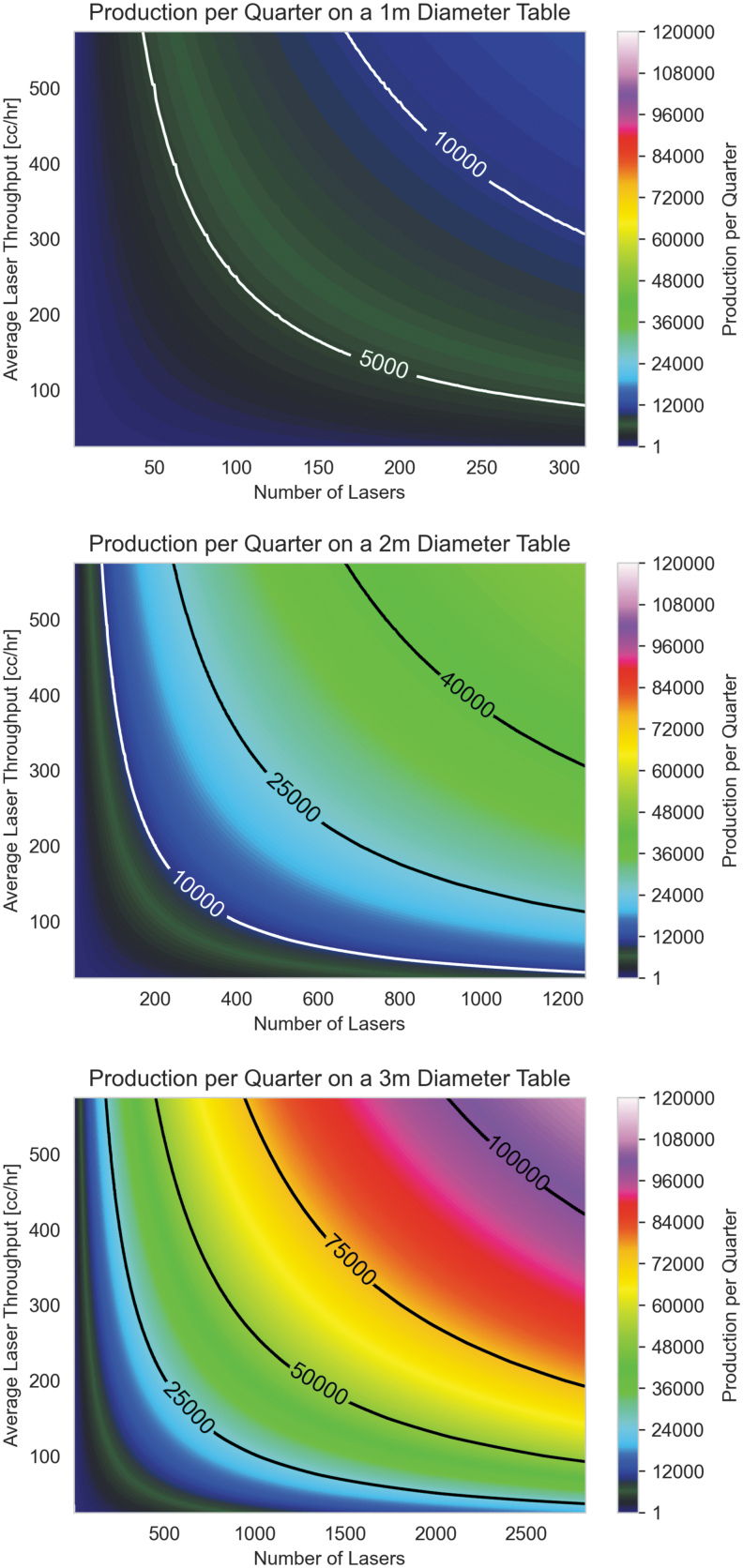
Quarterly production for one, two, and three meter diameter Rotary beds.

Figure 9 depicts the simulation of the quarterly output of rotary bed systems, with the top graph representing the potential for the existing system with a varying number of lasers and the bottom showing the most optimized case of a large toroidal bed and many lasers. The quarterly production rate in this well-optimized system exceeds 100,000 and is four orders of magnitude higher than current Cartesian LPBF systems available commercially at the time of writing of this article and are sufficient to provide current production levels necessary for the electric vehicle market with a small number of additive manufacturing systems.

Large toroidal build plates with many supported parts would be a difficult challenge for high-production-rate Rotary systems and will require additional infrastructure. However, the swapping of build plates (built vs. empty) could be completed rapidly and allow for any post-processing to be finished in parallel with printing. Consequently, post-processing would not materially affect the production throughput and is not considered in this analysis. Furthermore, the analysis relies on the analogy of Moore's Law for semiconductors, which are multifaceted (i.e., smaller chips, more transistors, less energy per function, faster operation, etc.), which we are assuming will improve maintenance costs for lasers. Energy costs are expected to be conservatively linear with the production rate so that the energy per part will remain constant. However, more likely, energy per part will be improved with increases in process efficiencies.

This output is dramatically improved relative to large Cartesian systems—the productivity of which was shown in Figure 8 for comparison. In addition, improvements in laser throughput or the number of lasers have a near-linear impact on system productivity and future developments for both are expected to improve exponentially in a similar manner to Moore's Law in the semiconductor industry.

By leveraging the capabilities of a rotary bed system and potential future improvements in laser and optics technology, production output comparable to traditional technologies with less waste and higher performance parts is possible. Using Eqs. (2–4), theoretical production values for a rotary bed system can be calculated. Using the same cylindrical sample motor and Table 5 shows potential configurations that would enable the production of 30K and 100K parts per quarter per system. The Optics Area in the table represents the area above the bed consumed by the optics train and the maximum number of lasers potentially used for a given table diameter.

**Table 5.** Future Production Values for Rotary Powder Bed Fusions with a Number of Hypothetical Lasers

Parts/quarter	Table diameter (m)	Optics area (cm^2^)	Laser productivity (cc/h)	Number of lasers
30K	3.0	125	300	565
	3.5	150	250	641
	4.0	125	150	718
100K	4.0	125	800	1005
	4.0	75	450	1675
	4.5	200	400	1590

In the economics of (the time of the writing of this article), based on Figure 3, the estimated cost per a 400 W laser system is ∼$2000; a Rotary PBF printer with 1000 lasers would include $2M of additional laser expenses; however, the assumption of work is that the cost of lasers continues to follow the exponential trend of Figure 3 (similar to Moore's Law in semiconductors), and is therefore assumed to exponentially decrease. We are also assuming that most PBF system costs are at or less than $1M USD and that this cost will remain constant.

For consideration of cost per part, with the 1000 hypothetical system at $1M ($11K monthly per amoritization over 10 years) and a production rate of 100,000 per quarter (33,333 parts per month), each part would hypothetically cost $3. The current production at 18 parts per quarter would result in cost per part of roughly $2000—a dramatic reduction in part cost over time.

### Additional techniques to improve LPBF productivity

With the current laser technologies, hundreds of lasers will need to work together to reach mass production levels. To enable each laser to melt more powder and reduce the number of lasers needed, a few potential technologies can be harnessed, in addition to rotary bed. Beam wobbling is a technique in which the laser is wobbled rapidly in a small circular motion to spread the energy over a larger area—enabling higher travel rates and greater hatch spacing. Islam *et al.* determined that beam wobbling could increase the rate of area melting by 40–50% relative to a nonwobbling laser.^27^

Examples of reduction of specific scan execution times have been reported as much as 95%.^28^ This technique could significantly reduce the number of lasers needed to meet the production quota or increase overall production. Different beam shapes could also prove useful. Tumkur *et al.* researched Bessel beams and have shown that non-Gaussian beam shapes can reduce keyholing porosity,^2,29^ which could enable high-powered lasers to function effectively without the risk of introducing defects due to excessive energy input, further increasing laser productivity.

## Conclusions

This work has provided a comparison of conventional Cartesian PBF systems to rotary PBF systems in terms of mass production (100's of thousands of structures fabricated per quarter). As rotary systems improve the laser duty cycle and effectively enable the dispensing and selective laser of multiple layers of a structure simultaneously, dramatic potential exists to exponentially improve production rates, and consequently, broad adoption of the digital manufacturing by industry can be realized. Much of the analysis assumes a trend of reductions in cost and size of lasers capable of selective laser melting the alloys of interest, and thus explores benefits of Rotary PBF that will likely be manifested in the decades to come.

Major understandings highlighted by this analysis include the following:

- Conventional Cartesian systems can meet production requirements if multiple lasers are integrated into each system to reduce the laser melting time. However, recoating time is unavoidable, and as a result, production rate improvement with more and faster lasers in a given system is a limited solution.
- Low production rates of Cartesian systems could also be overcome by implementing hundreds of systems in parallel, but this is a prohibitively expensive solution in terms of real estate, maintenance costs, and workforce requirements.
- Rotary systems provide the benefit of improved laser duty cycle—effectively eliminating the time dedicated to recoating as powder dispensing occurs simultaneously and in parallel with laser melting. This benefit could result in a potential exponential improvement in productivity by increasing the laser and powder deposition stations.
- Rotary systems can build many layers simultaneously and the number of layers is currently limited only by the cost and size of laser optics, which are presumed to improve over time. The increase in simultaneous layer fabrication is forecast in the proposed analysis to significantly improve the productivity of PBF and could lead to true mass production with additive manufacturing or in mid-level production in which high-performance materials and complex geometries improve application performance.

## References

[1] King WE, Anderson AT, Ferencz RM, et al. Laser powder bed fusion additive manufacturing of metals; physics, computational, and materials challenges. Phys Rev Appl 2015;2(4):041304.

[2] Sun S, Brandt M, Easton M. Powder bed fusion processes: An overview. In: Laser Additive Manufacturing. (Brandt M. ed.) Woodhead Publishing: Sawston, Cambridge; 2017; pp. 55–77.

[3] Khorasani A, Gibson I, Veetil JK, et al. A review of technological improvements in laser-based powder bed fusion of metal printers. Int J Adv Manuf Technol 2020;108(1–2):191–209.

[4] Flores I, Kretzschmar N, Azman AH, et al. Implications of lattice structures on economics and productivity of metal powder bed fusion. Addit Manuf 2020;31:100947.

[5] Tenbrock C, Kelliger T, Praetzsch N, et al. Effect of laser-plume interaction on part quality in multi-scanner Laser Powder Bed Fusion. Addit Manuf 2021;38:101810.

[6] de Formanoir C, Paggi U, Colebrants T, et al. Increasing the productivity of laser powder bed fusion: Influence of the hull-bulk strategy on part quality, microstructure and mechanical performance of Ti-6AL-4V. Addit Manuf 2020;33:101129.

[7] Du Plessis A, Yelamanchi B, Fischer C, et al. Productivity enhancement of laser powder bed fusion using compensated shelled geometries and hot isostatic pressing. Adv Ind Manuf Eng 2021;100031.

[8] Standard. ISO/ASTM 52900: 2015 Additive manufacturing-General principles-terminology. ASTM F2792-10e1. 2012.

[9] Sing SL, Yeong WY. Laser powder bed fusion for metal additive manufacturing: Perspectives on recent developments. Virtual Phys Prototyp 2020;15(3):359–370.

[10] Hauser C, Sutcliffe C, Egan M, et al. Spiral growth manufacturing (SGM)—A continuous additive manufacturing technology for processing metal powder by selective laser melting. In: Solid Freeform Fabr Symp Proc; 2005.

[11] Yigit IE, Khan SA, Lazoglu I. Robotic additive turning with a novel cylindrical slicing method. Int J Adv Manuf Technol 2022;119(11–12):7641–7651.

[12] Munasinghe N, Paul G. Radial slicing for helical-shaped advanced manufacturing applications. Int J Adv Manuf Technol 2021;112(3–4):1089–1100.

[13] Yigit IE, Lazoglu I. Helical slicing method for material extrusion-based robotic additive manufacturing. Progr Addit Manuf 2019;4(3):225–232.

[14] Avdeev A, Shvets A, Gushchin I, et al. Strength increasing additive manufacturing fused filament fabrication technology, based on spiral toolpath material deposition. Machines 2019;7(3):57.

[15] Tang Q, Yin S, Zhang Y, et al. A tool vector control for laser additive manufacturing in five-axis configuration. Int J Adv Manuf Technol 2018;98(5–8):1671–1684.

[16] Carter WT, Graham ME, Hayden CJ, et al. A large format DMLM system using a continuously rotating powder bed. Addit Manuf 2020;31:100983.

[17] Available from: CaliforniaEnergyCommission.ZeroEmissionsVehicleSalesinCaliforniaandUSA.https://electrek.co/2022/04/28/us-electric-car-sales-jumped-record-high-last-quarter/ [Last accessed: May 19, 2023].

[18] Ibrahim M, Bernier F, Lamarre JM. Design of a PM-assisted synchronous reluctance motor utilizing additive manufacturing of magnetic materials. In: 2019 IEEE Energy Conversion Congress and Exposition; pp. 1663–1668.

[19] Martinsen MKSK. Laser diodes: The power of brilliance—The past and future of high-power semiconductor lasers. Laser Focus World [Internet]; 2018.

[20] Carter W, Tucker M, Mahony M, et al. An open-architecture multi-laser research platform for acceleration of large-scale additive manufacturing (ALSAM). In: Solid Freeform Fabr Symp Proc; 2020; p. 7.

[21] Roschli A, Messing A, Borish M, et al. ORNL Slicer 2: A novel approach for additive manufacturing tool path planning. In: Solid Freeform Fabr Symp Proc; 2017; p. 7.

[22] Roschli A, Borish M, Barnes A, et al. ORNL Slicer 2—Open source copyright. Computer software. Available from: https://github.com/ORNLSlicer/Slicer-2.Web.

[23] Yavari R, Riensche A, Tekerek E, et al. Digitally twinned additive manufacturing: Detecting flaws in laser powder bed fusion by combining thermal simulations with in-situ meltpool sensor data. Mater Des 2021;211:110167.

[24] Mohr G, Altenburg SJ, Hilgenberg K. Effects of inter layer time and build height on resulting properties of 316L stainless steel processed by laser powder bed fusion. Addit Manuf 2020;32:101080.

[25] Chaudry MA, Mohr G, Hilgenberg K. Experimental and numerical comparison of heat accumulation during laser powder bed fusion of 316L stainless steel. Progr Addit Manuf [Internet]; 2022.

[26] Yavari R, Smoqi Z, Riensche A, et al. Part-scale thermal simulation of laser powder bed fusion using graph theory: Effect of thermal history on porosity, microstructure evolution, and recoater crash. Mater Des 2021;204:109685.

[27] Islam N, Schanz J, Kolb D, et al. Improvement of surface quality and process area rate in selective laser melting by beam oscillation scan technique. J Mater Eng Perform 2021;30(7):5108–5117.

[28] Matthias S, Lucas M, Sivam S, et al. Using wobble based laser scanning techniques in additive manufacturing applications. Lasers Manufac Conf Proc 2019; pp. 1–13.

[29] Tumkur TU, Voisin T, Shi R, et al. Nondiffractive beam shaping for enhanced optothermal control in metal additive manufacturing. Sci Adv 2021;7(38):eabg9358. 34524849 10.1126/sciadv.abg9358 PMC8443179

